# Temporal and Bidirectional Association Between Blood Pressure Variability and Arterial Stiffness: Cross-Lagged Cohort Study

**DOI:** 10.2196/45324

**Published:** 2023-07-04

**Authors:** Zhiyuan Wu, Haiping Zhang, Yutao Wang, Zhiwei Li, Xia Li, Lixin Tao, Xiuhua Guo

**Affiliations:** 1 School of Public Health Capital Medical University Beijing China; 2 Center of Precision Health Edith Cowan University Perth Australia; 3 Shanghai Fufan Information Technology Ltd Co Shanghai China; 4 Department of Mathematics and Statistics La Trobe University Melbourne Australia

**Keywords:** blood pressure variability, brachial-ankle pulse wave velocity, arterial stiffness, cross-lagged panel analysis

## Abstract

**Background:**

The causal relationship between blood pressure variability (BPV) and arterial stiffness remains debated.

**Objective:**

This study aimed to explore the temporal and bidirectional associations between long-term BPV and arterial stiffness using a cohort design with multiple surveys.

**Methods:**

Participants from the Beijing Health Management Cohort who underwent health examinations from visit 1 (2010-2011) to visit 5 (2018-2019) were enrolled in this study. Long-term BPV was defined as intraindividual variation using the coefficient of variation (CV) and SD. Arterial stiffness was measured by brachial-ankle pulse wave velocity (baPWV). The bidirectional relationship between BPV and arterial stiffness was explored using cross-lagged analysis and linear regression models, with records before and after visit 3 categorized as phase 1 and phase 2, respectively.

**Results:**

Of the 1506 participants, who were a mean of 56.11 (SD 8.57) years old, 1148 (76.2%) were male. The cross-lagged analysis indicated that the standardized coefficients of BPV at phase 1 directing to the baPWV level at phase 2 were statistically significant but not vice-versa. The adjusted regression coefficients of the CV were 4.708 (95% CI 0.946-8.470) for systolic blood pressure, 3.119 (95% 0.166-6.073) for diastolic pressure, and 2.205 (95% CI 0.300-4.110) for pulse pressure. The coefficients of the SD were 4.208 (95% CI 0.177-8.239) for diastolic pressure and 4.247 (95% CI 0.448-8.046) for pulse pressure. The associations were predominant in the subgroup with hypertension, but we did not observe any significant association of baPWV level with subsequent BPV indices.

**Conclusions:**

The findings supported a temporal relationship between long-term BPV and arterial stiffness level, especially among people with hypertension.

## Introduction

Hypertension is one of the most significant risk factors for heart failure, atrial fibrillation, stroke, chronic kidney disease, and overall cardiovascular disease (CVD), with a largely increasing health burden [1,2]. According to the Chinese Hypertension Survey, between 2012 and 2015, the prevalence of hypertension was 23.2% (approximately 244.5 million) among adults and over 55% in people aged 65 and older [3], imposing a significant life loss [4,5]. Arterial stiffness is an important pathological mechanism of high blood pressure that leads to various adverse outcomes [4,6]. Arterial stiffness, as an independent predictor of CVD, could cause microvascular damage to organs rich in low-resistance vascular tissue (kidney, brain, etc), affecting the myocardium and vascular function through afterload [7,8]. Both arterial stiffness and blood pressure are aggravated with aging and can lead to cardiovascular events. In addition, dietary and lifestyle factors are also important causes of arterial stiffness progression, and studies have shown that a plant-based diet can significantly reduce arterial stiffness and blood pressure levels [9-14]. Therefore, the precise identification of arterial stiffness risk markers for primary prevention and personalized treatment based on modifiable factors is essential to prevent the progression of arterial stiffness and reduce the risk of CVD.

Previous studies have shown that elevated blood pressure precedes arterial stiffness [15]. In addition to blood pressure, blood pressure variability (BPV), such as visit-to-visit BPV, day-to-day home BPV, and 24-hour ambulatory BPV, is related to CVD [16-19]. Numerous studies have shown that all types of BPV are positively associated with adverse cardiovascular events (coronary heart disease, stroke, CVD, mortality, etc) in people with hypertension [20-22], indicating the potential predictive capacity of BPV for an unfavorable arterial stiffness level. On the other hand, several studies have shown that age-dependent arterial stiffness could also cause exaggerated BPV [23,24]. An assessment of the temporal association between BPV and arterial stiffness can facilitate the early identification of cardiovascular risk and subsequent intervention [25]. However, the cross-sectional design of most existing studies cannot indicate the temporal relationship between BPV and arterial stiffness [24,26,27]; thus, the causal association between BPV and arterial stiffness needs further research [28].

In this study, we aimed to comprehensively investigate the temporal and bidirectional relationship between long-term BPV and arterial stiffness using a cross-lagged panel design and further explore the interaction effect of hypertension status in a large cohort.

## Methods

### Study Design and Population

This study included participants from the Beijing Health Management Cohort, which is a large-scale longitudinal cohort study investigating the risk factors and biomarkers of cardiometabolic disorders. Our study is a secondary analysis of the Beijing Health Management Cohort under a longitudinal cohort design with multiple consecutive surveys. In total, 2381 individuals aged 18 years or above underwent multiple comprehensive biennial health examinations at visit 1 (2010-2011), visit 2 (2012-2013), visit 3 (2014-2015), visit 4 (2016-2017), and visit 5 (2018-2019), and arterial stiffness data were collected. We excluded 760 individuals with missing blood pressure data at any health visit. To minimize the possible effect of reverse causality, 115 participants with CVD (coronary heart disease, myocardial infarction, and stroke) at visit 3 (as the baseline) were further excluded. Finally, this study was restricted to a subset of 1506 participants with complete data. The period from visit 1 to visit 3 was defined as phase 1, and the period from visit 3 to visit 5 was defined as phase 2, as illustrated in Figure S1 of Multimedia Appendix 1.

### Ethics Approval

This study was approved by the Ethics Committee of Capital Medical University (2020SY031). The study data were anonymous. All participants provided written informed consent before taking part in this study.

### Measurement and Data Collection

The participants’ sociodemographic characteristics and lifestyle and health-related information were collected via a standard questionnaire by our trained staff, including age, sex, education, smoking status, drinking status, regular physical activity, diagnosis history of diseases, and medication information. Educational level was categorized as no or some primary school, middle school or high school, and bachelor’s degree or above. Smoking was defined as “current,” “former,” and “never.” Drinking status was defined as “currently drinking” and “not currently drinking.” Regular physical activity was defined as having moderate or intense activity at work or during leisure time more than 4 times and 80 minutes per week. Vigorous activities make breathing much harder than normal and include heavy lifting, digging, plowing, aerobics, fast bicycling, and cycling with a heavy load. Moderate activities make breathing somewhat harder than normal and include carrying light loads, bicycling at a regular pace, mopping the floor, doing Taiji, and walking briskly. Mild activity refers to waking at work, at home, walking to travel from place to place, and any other walking that one might do solely for recreation, sport, exercise, or leisure [29].

The physical and biochemical examination data were acquired from the electronic medical record system. BMI was calculated as weight (in kg)/height (in meters squared). Systolic blood pressure (SBP) and diastolic blood pressure (DBP) were presented as the average of 2 measurements on the right arm using a sphygmomanometer after resting for at least 10 minutes. The pulse pressure (PP) was calculated as SBP minus DBP. Hypertension status was defined as SBP ≥140 mmHg or DBP ≥90 mmHg or use of any antihypertensive medication or self-reported history of hypertension according to the Seventh Report of the Joint National Committee on Prevention, Detection, Evaluation, and Treatment of High Blood Pressure (JNC-7) criteria [30]. Data on serum total cholesterol (TC), triglyceride, high-density lipoprotein cholesterol (HDL-C), low-density lipoprotein cholesterol (LDL-C), fasting blood glucose (FBG), glycated hemoglobin (HbA1c), and plasma high-sensitivity C-reactive protein (hs-CRP) were also collected in this study. Diabetes was defined as FBG ≥7.0 mmol/l, HbA1c ≥6.5%, or the use of any glucose-lowering medication or self-reported history of diabetes according to the American Diabetes Association [31]. Moreover, according to the Guidelines on Prevention and Treatment of Dyslipidemia for Chinese Adults [32], dyslipidemia was defined as triglyceride ≥2.3 mmol/l, TC ≥6.2 mmol/l, LDL-C ≥4.1 mmol/l, HDL-C <1.0 mmol/l, any lipid-lowering medication or self-reported history of dyslipidemia.

### Assessment of BPV and Arterial Stiffness

BPV was assessed at phase 1 (visit 1 to visit 3) and phase 2 (visit 3 to visit 5). Long-term BPV was defined as the intraindividual variation in SBP, DBP, and PP at 3 visits and measured by the coefficient of variation (CV) and SD. The mean value of 3 blood pressure indices at each phase was also calculated.

The arterial stiffness level was measured by brachial-ankle pulse wave velocity (baPWV), which is a simple, noninvasive, automatic method widely used in population-based studies. The baPWV was measured with an Omron Colin BP-203RPE III device (Omron Health Care). The time interval between the wave front of the brachial waveform and the ankle waveform is expressed as the time interval between the brachium and ankle (∆Tba). La indicates the path length from the suprasternal notch to the ankle, and Lb refers to the path length from the suprasternal notch to the brachium based on the height of the subjects. The final baPWV was calculated as (La-Lb)/∆Tba, which has been previously described [33]. The arterial stiffness level was defined as the mean of baPWV measurements at phases 1 and 2.

### Statistical Analyses

Baseline characteristics were presented as the mean (SD), median (IQR P_25_-P_75_), or number (percentage), as appropriate. We used the cross-lagged panel model to analyze the temporal relationship between BPV and baPWV levels. The lagged effect between the observed variables, the correlation between the variables at each phase, and the residual variance of the observed variables were estimated in the cross-lagged panel analysis, which elucidates the temporal relationship between correlated variables and indicates the precursor variable [34,35].

Then, a multivariable linear regression model was used to estimate the bidirectional associations between BPV and baPWV levels. We calculated the regression coefficients of BPV indices at phase 1, with baPWV at phase 2 as the independent variable, and the coefficients of baPWV level at phase 1, with BPV indices at phase 2 as the independent variable. To adjust for potential confounding factors, three models were established: (1) model 1 was adjusted for the individual-level mean blood pressure; (2) model 2 was adjusted for mean blood pressure, age, and sex; and (3) model 3 was further adjusted for BMI, education level, smoking, drinking, physical activity, hypertension, dyslipidemia, diabetes, triglyceride, TC, FBG, HbA1c, and hs-CRP. In total, there were 17 variables in the regression model. Thus, a group of 1506 participants in this study could satisfy the sample size requirement given the principle of 10 samples per variable in the regression model [36]. Furthermore, the blood pressure at visit 3 was adjusted in the models in place of the mean blood pressure value in the sensitivity analysis. The difference was considered statistically significant at 2-sided *P*<.05, and a 95% CI was provided.

In addition, we analyzed the association of BPV with arterial stiffness levels in the subgroups with and without hypertension. The use of antihypertensive medication was additionally adjusted for in the subgroup with hypertension, considering that antihypertensive agents are an important determinant of BPV and are related to arterial stiffness levels. The difference was considered statistically significant at 2-sided *P*<.1, and a 90% CI was provided in the subgroup analysis [37].

All the analyses were conducted using R software (version 4.1.0; R Foundation for Statistical Computing), using the packages *lavaan* for cross-lagged panel analysis and *ggplot2* for visualization.

## Results

### Characteristics

Among the 1506 participants, the mean age was 56.11 (SD 8.57) years, and 661 (43.9%) were diagnosed with hypertension at visit 3. The median value of the cumulative average level of baPWV was 1395.50 (IQR 1298.12-1544.34) cm/s at phase 1 and 1438.25 (IQR 1344.00-1588.67) cm/s at phase 2. Detailed characteristics of the study population and the variability indices of blood pressure are summarized in Tables 1 and 2, respectively.

**Table 1:** Characteristics of the study population (N=1506) at baseline (visit 3).

Characteristics		Variables
Age (years), mean (SD)		56.11 (8.57)
**Sex, n (%)**		
	Male	1148 (76.2)
	Female	358 (23.8)
BMI^a^ (kg/m^2^), mean (SD)		25.67 (2.90)
**Education level, n (%)**		
	Primary	122 (8.1)
	Secondary	794 (52.7)
	Postsecondary	590 (39.2)
Physical activity, n (%)		751 (49.9)
**Smoking, n (%)**		
	None	851 (56.5)
	Former	218 (14.5)
	Current	437 (29.0)
Current drinking, n (%)		931 (61.8)
Hypertension, n (%)		661 (43.9)
Antihypertensive medication, n (%)		507 (33.7)
Diabetes, n (%)		250 (16.6)
Dyslipidemia, n (%)		667 (44.3)
SBP^b^ (mmHg), mean (SD)		122.09 (14.59)
DBP^c^ (mmHg), mean (SD)		71.05 (9.68)
Fasting plasma glucose^d^ (mmol/L), mean (SD)		5.58 (1.04)
Glycated hemoglobin, mean (SD)		5.71 (0.63)
Triglyceride^d^ (mmol/L), mean (SD)		1.63 (0.99)
TC^d^ (mmol/L), mean (SD)		4.80 (0.86)
hs-CRP^e^ (mg/L), median (IQR)		0.69 (0.28-1.27)

^a^Calculated as weight in kilograms divided by height in meters squared.

^b^SBP: systolic blood pressure.

^c^DBP: diastolic blood pressure.

^d^TC: total cholesterol. To convert fasting plasma glucose to mg/dl, multiply by 18; to convert triglyceride to mg/dl, multiply by 88.6; to convert TC to mg/dl, multiply by 38.66.

^e^hs-CRP: high-sensitivity C-reactive protein.

**Table 2:** BPV^a^ and arterial stiffness parameters.

BPV measurements	Phase 1	Phase 2
CV^b^ of SBP^c^, median (IQR)	5.57 (2.99-8.74)	5.81 (3.51-8.85)
CV of DBP^d^, median (IQR)	7.06 (3.67-10.95)	6.60 (3.98-10.18)
CV of PP^e^, median (IQR)	10.88(6.02-16.62)	10.70(6.29-15.71)
SD of SBP, median (IQR)	7.00 (3.54-10.99)	7.07 (4.24-10.61)
SD of DBP, median (IQR)	4.95 (2.83-7.98)	4.62 (2.83-7.09)
SD of PP, median (IQR)	5.29(2.83-8.14)	5.29(3.06-7.78)
baPWV^f^ level, median (IQR), cm/s	1395.50 (1298.12-1544.34)	1438.25 (1344.00-1588.67)

^a^BPV: blood pressure variability.

^b^CV: coefficient of variation.

^c^SBP: systolic blood pressure.

^d^DBP: diastolic blood pressure.

^e^PP: pulse pressure.

^f^baPWV: brachial-ankle pulse wave velocity.

### Temporal Relationship Between BPV and baPWV

**Figure 1:**
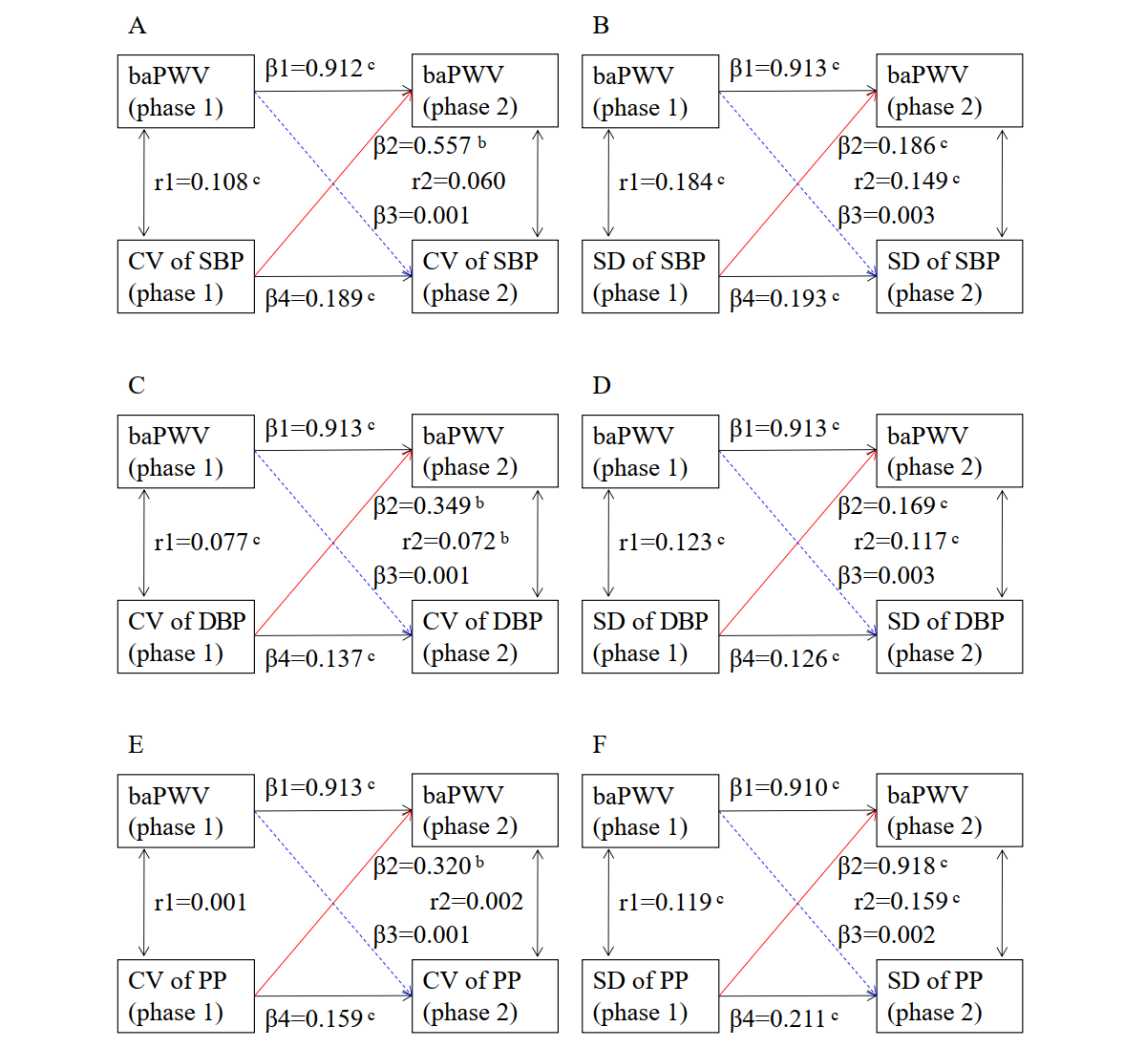
Bidirectional analysis of blood pressure variability (BPV) indices and brachial-ankle pulse wave velocity (baPWV) level by cross-lagged panel analysis. β1: baPWV at phase 1→baPWV at phase 2; β2: BPV at phase 1→baPWV at phase 2; β3: baPWV at phase 1→BPV at phase 2; β4: BPV at phase 1→BPV at phase 2; r1: correlation coefficient between BPV and baPWV at phase 1; r2: correlation coefficient between BPV and baPWV at phase 2. A: BPV was measured by coefficient of variation (CV) of systolic blood pressure (SBP); B: BPV was measured by SD of SBP; C: BPV was measured by CV of diastolic blood pressure (DBP); D: BPV was measured by SD of DBP; E: BPV was measured by CV of pulse pressure (PP); F: BPV was measured by SD of PP. a means *P*<.05; b means *P*<.01; c means *P*<.001.

Assessments of BPV indices and baPWV at phase 1 and phase 2 constituted a typical cross-lagged panel design. Figure 1 presents the association of BPV indices at phase 1 with baPWV level at phase 2 and the association of baPWV at phase 1 with the BPV indices at phase 2. The standardized coefficients of the CV of SBP, DBP, and PP were 0.557, 0.349, and 0.320, respectively, and the SDs of SBP, DBP, and PP were 0.186, 0.169, and 0.918, respectively, to the follow-up baPWV level (all *P*<.01), while the standardized coefficients of baPWV level at phase 1 to the subsequent BPV indices were all weak and insignificant.

### Bidirectional Relationship Between BPV and baPWV

In the fully adjusted model (model 3), a 1-unit increase in the CV of SBP, DBP, and PP at phase 1 was associated with a 4.708 (95% CI 0.946-8.470), 3.119 (95% CI 0.166-6.073), and 2.205 (95% CI 0.300-4.110) increase, respectively, in the baPWV level at phase 2. Similarly, a 1-unit increase in the SD of DBP and PP at phase 1 was associated with a 4.208 (95% CI 0.177-8.239) and 4.247 (95% CI 0.448-8.046) increase in the baPWV level at phase 2 (Table 3). The positive linear regression lines of the BPV indices at phase 1 and the baPWV level at phase 2 are presented in Figure S2 of Multimedia Appendix 1. In contrast, we did not observe any significant association between the baPWV level at phase 1 and the subsequent BPV indices (Table S1 of Multimedia Appendix 1).

**Table 3:** Association between BPV^a^ at phase 1 and the cumulative average level of baPWV^b^ at phase 2^c^.

BPV measurements	Model 1^d^				Model 2^e^					Model 3^f^		
	Coefficient	95% CI	*P* value	Coefficient		95% CI		*P* value	Coefficient		95% CI	*P* value
**SBP^g^ at phase 1**												
CV^h^	4.748	2.617 to 6.880	<.001	2.320		0.559 to 4.080	.01		4.708		0.946 to 8.470	.02
SD	3.714	2.001 to 5.428	<.001	1.800		0.385 to 3.215	.01		1.666		−0.870 to 4.202	.20
**DBP^i^ at phase 1**												
CV	4.571	2.662 to 6.480	<.001	−0.246		−1.721 to 1.230	.74		3.119		0.166 to 6.073	.04
SD	6.099	3.499 to 8.699	<.001	−0.133		−2.138 to 1.872	.90		4.208		0.177 to 8.239	.04
**PP^j^ at phase 1**												
CV	2.088	0.983 to 3.193	<.001	1.309		0.335 to 2.283	.009		2.205		0.300 to 4.110	.02
SD	4.416	2.234 to 6.599	<.001	2.777		0.852 to 4.703	.005		4.247		0.448 to 8.046	.03

^a^BPV: blood pressure variability.

^b^baPWV: brachial-ankle pulse wave velocity.

^c^Phase 1 refers to the period from visit 1 to visit 3, and phase 2 refers to the period from visit 3 to visit 5.

^d^Adjusted for the mean blood pressure level during phase 1.

^e^Adjusted for the mean blood pressure level during phase 1, age, and sex.

^f^Adjusted for BMI, education level, smoking, drinking, physical activity, hypertension or not, dyslipidemia or not, diabetes or not, triglyceride, total cholesterol (TC), fasting glucose, glycated hemoglobin (HbA1c), and high-sensitivity C-reactive protein (hs-CRP), in addition to the covariates in model 2.

^g^SBP: systolic blood pressure.

^h^CV: coefficient of variation.

^i^DBP: diastolic blood pressure.

^j^PP: pulse pressure.

### Sensitivity Analysis

The results remained almost consistent when the blood pressure level at visit 3 was alternatively adjusted in the model, apart from PP (Table S2 of Multimedia Appendix 1). Spearman correlation coefficients among CV, SD, mean, maximum, and minimum SBP, DBP, and PP at the 2 phases are shown in Table S3 in Multimedia Appendix 1. There were strong correlations between the CV and SD of blood pressure indices and weak correlations of BPV indices with the mean, maximum, and minimum blood pressure, which indicated that BPV indices could reflect a novel feature beyond blood pressure itself.

### Subgroup Analysis

**Figure 2:**
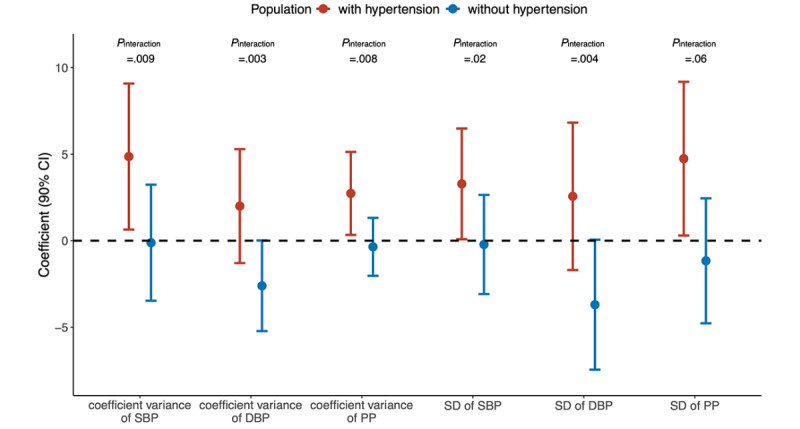
Associations of blood pressure variability (BPV) indices and the subsequent artery stiffness level in participants with both hypertension and normal blood pressure.The coefficient was adjusted for the mean blood pressure, age, sex, BMI, education level, smoking, drinking, physical activity, hypertension or not, dyslipidemia or not, diabetes or not, triglyceride, total cholesterol, fasting glucose, glycated hemoglobin (HbA1c), hs-CRP (high-sensitivity C-reactive protein), and antihypertensive medication use (in participants with hypertension). DBP: diastolic blood pressure; PP: pulse pressure; SBP: systolic blood pressure.

We found that the relationship between BPV indices at phase 1 and baPWV level at phase 2 was affected by hypertension status (Figure 2). The CV and SD of SBP and PP were suggested to be associated with the subsequent baPWV level in participants with hypertension. A 1-unit increase in the CV of SBP and PP at phase 1 was associated with a 4.861 (90% CI 0.642-9.081) and 2.733 (90% CI 0.335-5.131) increase in baPWV level at phase 2, respectively, and a 1-unit increase in the SD of SBP and PP was associated with a 3.283 (90% CI 0.083-6.484) and 4.740 (90% CI 0.299-9.182) increase in baPWV, respectively. Table 4 summarizes the impact of the mean blood pressure, BPV indices, and antihypertensive medication (only in participants with hypertension) on the follow-up baPWV level. The direct blood pressure level was positively associated with the baPWV level in participants with both hypertension and normal blood pressure, and the use of antihypertensive medication was significantly associated with a lowered baPWV level in the subgroup with hypertension.

**Table 4:** Association of BPV^a^, mean blood pressure, and use of antihypertensive medication with the subsequent baPWV^b^ levels among participants with both hypertension and normal blood pressure^c^.

Measurements	Hypertension			Normal blood pressure		
	Coefficient^d^	90% CI	*P* value	Coefficient^d^	90% CI	*P* value
CV^e^ of SBP^f^	4.861	0.642 to 9.081	.06	−0.117	−3.471 to 3.237	.95
Mean of SBP	4.273	2.651 to 5.895	<.001	4.554	3.227 to 5.882	<.001
Antihypertensive medication	−54.524	−102.105 to −6.944	.06	N/A^g^	N/A	N/A
SD of SBP	3.283	0.083 to 6.484	.09	−0.219	−3.083 to 2.645	.90
Mean of SBP	4.029	2.373 to 5.685	<.001	4.569	3.227 to 5.911	<.001
Antihypertensive medication	−54.617	−102.292 to −6.942	.06	N/A	N/A	N/A
CV of DBP	2.000	−1.293 to 5.293	.32	−2.609	−5.224 to 0.013	.11
Mean of DBP	4.653	2.078 to 7.227	.003	6.208	4.104 to 8.313	<.001
Antihypertensive medication	−62.483	−111.44 to −13.525	.04	N/A	N/A	N/A
SD of DBP^h^	2.564	−1.698 to 6.825	.32	−3.698	−7.452 to 0.056	.11
Mean of DBP	4.411	1.762 to 7.059	.007	6.492	4.335 to 8.649	<.001
Antihypertensive medication	−62.168	−111.086 to −13.25	.04	N/A	N/A	N/A
CV of PP^i^	2.733	0.335 to 5.131	.06	−0.353	−2.028 to 1.321	.73
Mean of PP	4.116	1.964 to 6.268	.002	4.505	2.45 to 6.56	<.001
Antihypertensive medication	−56.565	−105.21 to −7.921	.06	N/A	N/A	N/A
SD of PP	4.740	0.299 to 9.182	.08	−1.162	−4.774 to 2.449	.60
Mean of PP	3.492	1.319 to 5.665	.009	4.603	2.569 to 6.638	<.001
Antihypertensive medication	−57.520	−106.175 to −8.865	.049	N/A	N/A	N/A

^a^BPV: blood pressure variability.

^b^baPWV: brachial-ankle pulse wave velocity.

^c^The associations between BPV indices and baPWV measurement were examined in separate regression models.

^d^Model was adjusted for the mean blood pressure, age, sex, BMI, education level, smoking, drinking, physical activity, hypertension or not, dyslipidemia or not, diabetes or not, triglyceride, total cholesterol (TC), fasting glucose, HbA1c, hs-CRP, and antihypertensive medication use (in participants with hypertension).

^e^CV: coefficient of variation.

^f^SBP: systolic blood pressure.

^g^N/A: not applicable.

^h^DBP: diastolic blood pressure.

^i^PP: pulse pressure.

## Discussion

### Principal Findings

This study explored the temporal and bidirectional associations between long-term visit-to-visit BPV and arterial stiffness levels based on a Chinese general population. In the cross-lagged analyses, the BPV indices were shown to have a temporal relationship with subsequent arterial stiffness, indicating that BPV is an independent predictor of unfavorable arterial stiffness levels and precedes arteriosclerosis progression, especially in people with hypertension. The classification of the temporal relationship in this study provides a scientific basis for identifying individuals at a high risk of arterial stiffness, further offering opportunities to advance primary prevention and personalized interventions for arterial stiffness.

Blood pressure usually stabilizes within a certain range under the complex interaction of environmental factors and normal cardiovascular regulation function. Due to baroreflex dysfunction associated with arteriole remodeling, BPV, and arterial stiffness levels increase with age, but the mutual associations between BPV and arterial stiffness remain unclear [38]. Previous studies have shown that BPV, such as visit-to-visit BPV, day-by-day home BPV, or 24-hour ambulatory BPV, is an independent risk factor for CVD [24,39]. In a post hoc patient-level analysis of 7 clinical trials, Donald et al [40] assessed the association between visit-to-visit BPV and percent atheroma volume and found that in patients with medically treated coronary artery disease, higher BPV, especially systolic BPV, is significantly associated with atherosclerotic progression and adverse cardiovascular outcomes such as death, myocardial infarction, stroke, emergency revascularization for acute coronary syndrome, or hospitalization for unstable angina. In terms of short-term BPV, Sander et al [41] found that diurnal systolic BPV is a significant predictor of atherosclerosis progression in people with hypertension via long-term continuous monitoring of circadian BPV and measurement of atherosclerosis progression using common carotid artery intima-media thickness. In the progression of CVD, arterial stiffness is an important pathological basis and early lesion signal for adverse events [6]. Our study supplements the evidence about the temporal and bidirectional relationships between the visit-to-visit BPV and arterial stiffness level using a cross-lagged cohort design and indicates that BPV could precede arterial stiffness and arteriosclerosis, especially in people with hypertension.

The pathological mechanism between BPV and arterial stiffness or CVD has not been completely determined [17,28]. According to the previous literature, endothelial dysfunction caused by vascular remodeling plays an important role in BPV-induced arterial stiffness, subsequently leading to the development of CVD [28,42-44]. Keith et al [45] found that BPV is positively correlated with endothelial dysfunction, as reflected in a significant association between higher BPV with lower endothelium-dependent flow-mediated vasodilation (FMD) and lower FMD/non–endothelium-dependent nitroglycerin-mediated vasodilation (NMD) ratio, which suggests that endothelium-specific vasodilation is impaired in individuals with higher BPV. Moreover, a significant association between endothelial dysfunction and arterial stiffness has been observed in clinical studies. For example, Carmel et al [44] demonstrated that brachial artery FMD is negatively correlated with aortic pulse wave velocity (PWV) in healthy individuals, which implies that endothelial dysfunction mediates the effect of BPV on arterial stiffness.

Furthermore, the hemodynamic environment changes caused by BPV may be an important reason for vascular endothelial dysfunction, which is closely related to the progression of arterial stiffness and atherosclerotic diseases [42,46]. The shear stress (SS) generated by blood flow and vascular endothelium triggers the release of various vasoactive mediators from endothelial cells, among which nitric oxide (NO), produced by endothelial-type nitric oxide synthase (eNOS or NOS3), is considered the main endothelial diastolic factor [47]. Under physiological conditions, a dynamic balance is maintained between blood flow stimulation and endothelial cell responses to maintain a stable internal environment [47]. Stable blood flow results in higher endothelial SS, lower intimal hyperplasia, and reduced atherosclerosis risk [25,48]. In contrast, complicated blood flow patterns produce low endothelial SS in the vascular wall, which facilitates the transshipment and accumulation of atherosclerotic risk factors, such as low-density lipoprotein to the intima of the vascular wall, and facilitates the inflammatory response of the vascular wall [48,49]. In addition, oscillatory SS induced by complex flow patterns can induce the expression of leukocyte adhesion molecules in endothelial cells, recruit inflammatory cells (monocytes and T cells) to the arterial wall, and initiate local vascular inflammation [49,50]. Oscillatory SS could also promote the activation of the oxidation process by increasing nicotinamide adenine dinucleotide-reduced (NADH) oxidase activity [51]. In the physiological state, the continuous release of NO from endothelial cells contributes to the functional regulation of arterial elasticity, and the combined effect of oscillatory SS and pressure could downregulate the expression level of endothelial constitutive nitric oxide synthase (EcNOS) and reduce the synthesis of NO [52]. Increased BPV might adversely affect the stability of hemodynamics in a physiological setting, thereby affecting vascular endothelial function.

In addition, previous population-based studies have shown that high BPV is strongly associated with biomarkers of vascular inflammation, independent of the mean levels of blood pressure [53]. In short, BPV could be both a cause and an indicator of arterial stiffness and atherosclerosis through hemodynamic environment changes and vascular endothelial dysfunction. In another prospective cohort, researchers found that baseline common carotid arterial intima-media thickness was not significantly associated with BPV during follow-up [41], which suggests that BPV is the initial cause of arteriosclerosis and arterial stiffness from another aspect [42] and could be the underlying pathological mechanism and therapeutic target of arterial stiffness and related atherosclerotic vascular diseases. Interventions in diet, lifestyle habits, and pharmacological treatment in high-risk groups can effectively reduce arterial stiffness levels [54,55]. The temporal association between increased BPV and arterial stiffness identified in this study further emphasizes the role of BPV levels as an early warning for the risk of arterial stiffness. Our findings are potentially valuable for delaying or avoiding the progression of arterial stiffness at a population level and promoting early and active cardiovascular health in the public health setting.

Based on the vicious cycle of hemodynamic stress and vascular disease, Kario et al [24] proposed the concept of systemic hemodynamic atherothrombotic syndrome (SHATS). Patients were assessed with scales that synergistically consider hemodynamic stress (various forms of blood pressure and BPV) and vascular disease to identify SHATS early and provide effective intervention to reduce adverse cardiovascular events and target organ damage [24]. In this study, we further demonstrated the potential role of maintaining blood pressure stability and reducing BPV to control arterial stiffness status. On the one hand, we found that the use of antihypertensive medication is associated with decreased arterial stiffness levels. Combination therapy with long-acting calcium channel blockers and angiotensin II receptor blockers has demonstrated to be effective in reducing both long-term BPV and arterial stiffness [56]. On the other hand, the association between BPV and arterial stiffness is still significant after adjusting for the use of antihypertensive medication. Previous studies on antihypertensive medication adherence also found that low adherence resulted in only a small increase in BPV compared with high adherence, and BPV was significantly associated with CVD independent of medication adherence [57,58], which underlines the comprehensive and novel strategy for controlling BPV levels. Strict BP control by taking medication, together with leading a healthy lifestyle, may play a pivotal role in suppressing arterial stiffening via BPV reduction in people with hypertension [59]. 

In addition, this study found that BPV preceded arterial stiffness, suggesting a high risk of CVD in people with higher BPV. Previous studies have shown that plant-based diets have protective effects against CVD. In particular, the Mediterranean diet significantly reduces arterial stiffness and blood pressure levels [9], and Dietary Approaches to Stop Hypertension (DASH) combined with exercise can significantly reduce blood pressure in people with intractable hypertension [13,14]. Additionally, moderate nut intake can also help control CVD risk factors such as weight by reducing appetite and fat absorption [11]. Enhanced monitoring of BPV and individualized interventions in diet, lifestyle habits, and pharmacotherapy for those at a high risk of increased BPV are significant in reducing the risk of arterial stiffness and the occurrence of subsequent cardiovascular events [60-62]. Based on these study findings, the integration of long-term monitoring of blood pressure levels into community-level health examinations is essential to optimize the prevention efficacy of CVD. Long-term BPV complements the performance of early CVD risk assessment at the population level compared to a single-point level of blood pressure, providing an opportunity to shift the focus from treatment to population-level risk prevention, which emphasizes the potential effectiveness of community-level continuous monitoring of blood pressure levels and early identification of increased BPV. Among people with hypertension, enhancing medication adherence and adhering to healthy dietary habits to maintain stable blood pressure over a long period are important for preventing subsequent adverse cardiovascular outcomes.

### Limitations

Several limitations of this study should be acknowledged. First, different antihypertensive drugs have distinct effects on BPV and arterial stiffness levels, but detailed information on medication use is lacking in our study. The interaction effect between different antihypertensive medications and BPV on arterial stiffness levels needs further investigation. Second, this study is based on a Chinese cohort, and the observed results warrant further validation in other populations with large sample sizes. Data on household socioeconomic status and food intake were not collected in the cohort. The estimated effect size could be biased given the potential effects of socioeconomic status and dietary patterns on the arterial stiffness level. Third, the specific effect of maintaining long-term stable blood pressure on suppressing arterial stiffening and promoting cardiovascular health warrants further research.

### Conclusions

Our findings suggested a temporal relationship between visit-to-visit BPV and arterial stiffness during 10-year surveys, especially in people with hypertension. Maintaining long-term stable blood pressure should be emphasized for suppressing arterial stiffness. This study further demonstrates that long-term BPV provides a new target for the early prevention of CVD in the public health setting, suggesting the potential benefit of incorporating long-term monitoring and assessment of BPV into community-level health examinations.

## Appendix

Multimedia Appendix 1 Supplemental tables and figures.

## Abbreviations

∆Tba: time interval between the brachium and ankle
baPWV: brachial-ankle pulse wave velocity
BPV: blood pressure variability
CV: coefficient of variation
CVD: cardiovascular disease
DASH: Dietary Approaches to Stop Hypertension
DBP: diastolic pressure
EcNOS: endothelial constitutive nitric oxide synthase
eNOS: endothelial-type nitric oxide synthase
FBG: fasting blood glucose
FMD: flow-mediated vasodilation
HbA1c: glycated hemoglobin
HDL-C: high-density lipoprotein cholesterol
hs-CRP: high-sensitivity C-reactive protein
JNC-7: Seventh Report of the Joint National Committee on Prevention, Detection, Evaluation, and Treatment of High Blood Pressure
LDL-C: low-density lipoprotein cholesterol
NADH: nicotinamide adenine dinucleotide-reduced
NMD: non–endothelium-dependent nitroglycerin-mediated vasodilation
NO: nitric oxide
PWV: pulse wave velocity
SBP: systolic blood pressure
SHATS: systemic hemodynamic atherothrombotic syndrome
SS: shear stress
TC: total cholesterol

## Data Availability

Data collected for this study are available on request with publication; requests should be directed to the corresponding author.
