# Supplementary material for: Temporal and Bidirectional Association Between Blood Pressure Variability and Arterial Stiffness: Cross-Lagged Cohort Study
Source: JMIR Public Health Surveill. 2023 Jul 4;9:e45324. doi: 10.2196/45324 (PMC10354655; doi:10.2196/45324)
Supplement: Multimedia Appendix 1 [file publichealth_v9i1e45324_app1.doc]

**Supplement Material**

**Temporal and bidirectional association between blood pressure variability and arterial stiffness: a cross-lagged cohort study**

Zhiyuan Wu et al.

**1 Supplement Tables:**

**Table S1:** Association from the cumulative average level of baPWV at phase 1 to the variation of blood pressure at phase 2.

**Table S2:** Association from the variation of blood pressure at phase 1 to the cumulative average level of baPWV at phase 2 when the blood pressure value at baseline adjusted.

**Table S3:** Correlation between coefficient variance, standard deviation, mean, maximum and minimum values of blood pressure at phase 1 and phase 2.

**2 Supplement Figures:**

**Figure S1:** Study period and definition of two phases.

**Figure S2:** The regression lines of BPV indexes at phase 1 and the subsequent artery stiffness level.

**Table S1**: Association from the cumulative average level of baPWV at phase 1 to the variation of blood pressure at phase 2.

|  | model | Coefficient | 95% CI | *P* value |
| --- | --- | --- | --- | --- |
| **dependent variable at phase 2** |  |  |  |  |
| coefficient variance of SBP | model 1 | 0.002 | 0.000 to 0.003 | .005 |
| coefficient variance of SBP | model 2 | 0.000 | -0.001 to 0.001 | .86 |
| coefficient variance of SBP | model 3 | -0.002 | -0.005 to 0.000 | .12 |
| standard deviation of SBP | model 1 | 0.003 | 0.001 to 0.004 | <.001 |
| standard deviation of SBP | model 2 | 0.001 | -0.001 to 0.002 | .57 |
| standard deviation of SBP | model 3 | -0.002 | -0.006 to 0.001 | .14 |
| coefficient variance of DBP | model 1 | 0.002 | 0.001 to 0.003 | .001 |
| coefficient variance of DBP | model 2 | 0.000 | -0.001 to 0.001 | .97 |
| coefficient variance of DBP | model 3 | -0.001 | -0.004 to 0.002 | .38 |
| standard deviation of DBP | model 1 | 0.001 | 0.000 to 0.002 | .006 |
| standard deviation of DBP | model 2 | 0.000 | -0.001 to 0.001 | .97 |
| standard deviation of DBP | model 3 | -0.001 | -0.003 to 0.001 | .28 |
| coefficient variance of PP | model 1 | 0.002 | 0.001 to 0.003 | .003 |
| coefficient variance of PP | model 2 | 0.000 | -0.002 to 0.001 | .50 |
| coefficient variance of PP | model 3 | -0.001 | -0.004 to 0.002 | .36 |
| standard deviation of PP | model 1 | 0.001 | 0.000 to 0.002 | .01 |
| standard deviation of PP | model 2 | 0.000 | -0.001 to 0.001 | .46 |
| standard deviation of PP | model 3 | -0.001 | -0.003 to 0.001 | .27 |

Abbreviations: SBP, systolic blood pressure; DBP, diastolic blood pressure; PP, pulse pressure; baPWV, brachial-ankle pulse wave velocity; CI, confidence interval.

model 1: Adjusted for the mean blood pressure level during phase 1; model 2: Adjusted for the mean blood pressure level during phase 1, age, sex; model 3: Adjusted for BMI, education level, smoking, drinking, physical activity, hypertension or not, dyslipidaemia or not, diabetes or not, triglyceride, total cholesterol, fasting glucose, HbA1c, and hs-CRP, in addition to covariates in model 2.

**Table S2**: Association from the variation of blood pressure at phase 1 to the cumulative average level of baPWV at phase 2 when the blood pressure value at baseline adjusted.

|  | model | Coefficient | 95% CI | *P* value |
| --- | --- | --- | --- | --- |
| **independent variable at phase 1** |  |  |  |  |
| coefficient variance of SBP | model 1 | 5.983 | 3.855 to 8.111 | <.001 |
| standard deviation of SBP | model 1 | 5.079 | 3.393 to 6.765 | <.001 |
| coefficient variance of DBP | model 1 | 5.698 | 3.763 to 7.632 | <.001 |
| standard deviation of DBP | model 1 | 7.822 | 5.269 to 10.375 | <.001 |
| coefficient variance of PP | model 1 | 0.986 | -0.125 to 2.097 | .08 |
| standard deviation of PP | model 1 | 3.232 | 1.006 to 5.458 | .004 |
| coefficient variance of SBP | model 2 | 3.233 | 1.484 to 4.981 | <.001 |
| standard deviation of SBP | model 2 | 2.786 | 1.4 to 4.173 | <.001 |
| coefficient variance of DBP | model 2 | 1.615 | 0.134 to 3.096 | .03 |
| standard deviation of DBP | model 2 | 2.760 | 0.807 to 4.712 | .006 |
| coefficient variance of PP | model 2 | 0.720 | -0.252 to 1.691 | .15 |
| standard deviation of PP | model 2 | 2.133 | 0.183 to 4.084 | .032 |
| coefficient variance of SBP | model 3 | 4.829 | 1.068 to 8.59 | .01 |
| standard deviation of SBP | model 3 | 2.009 | -0.488 to 4.506 | .12 |
| coefficient variance of DBP | model 3 | 3.119 | 0.157 to 6.082 | .04 |
| standard deviation of DBP | model 3 | 4.111 | 0.154 to 8.069 | .04 |
| coefficient variance of PP | model 3 | 1.421 | -0.479 to 3.32 | 0.14 |
| standard deviation of PP | model 3 | 3.600 | -0.226 to 7.426 | .07 |

Abbreviations: SBP, systolic blood pressure; DBP, diastolic blood pressure; PP, pulse pressure; baPWV, brachial-ankle pulse wave velocity; CI, confidence interval.

model 1: Adjusted for the blood pressure level at visit 3; model 2: Adjusted for the blood pressure level at visit 3, age, sex; model 3: Adjusted for BMI, education level, smoking, drinking, physical activity, hypertension or not, dyslipidaemia or not, diabetes or not, triglyceride, total cholesterol, fasting glucose, HbA1c, and hs-CRP, in addition to covariates in model 2.

**Table S3**: Correlation between coefficient variance, standard deviation, mean, maximum and minimum values of blood pressure at phase 1 and phase 2.

| with | variable | N | phase 1 |  | phase 2 |  |
| --- | --- | --- | --- | --- | --- | --- |
|  |  |  | Rho ^a^ | *P* value | Rho ^a^ | *P* value |
| sd of SBP |  |  |  |  |  |  |
|  | cv of SBP | 1506 | 0.989 | <.001 | 0.985 | <.001 |
|  | mean of SBP | 1506 | 0.217 | <.001 | 0.159 | <.001 |
|  | max of SBP | 1506 | 0.469 | <.001 | 0.430 | <.001 |
|  | min of SBP | 1506 | -0.093 | <.001 | -0.158 | <.001 |
| cv of SBP |  |  |  |  |  |  |
|  | mean of SBP | 1506 | 0.085 | .04 | 0.010 | .999 |
|  | max of SBP | 1506 | 0.347 | <.001 | 0.290 | <.001 |
|  | min of SBP | 1506 | -0.223 | <.001 | -0.304 | <.001 |
| sd of DBP |  |  |  |  |  |  |
|  | cv of DBP | 1506 | 0.985 | <.001 | 0.981 | <.001 |
|  | mean of DBP | 1506 | 0.188 | <.001 | 0.145 | <.001 |
|  | max of DBP | 1506 | 0.485 | <.001 | 0.406 | <.001 |
|  | min of DBP | 1506 | -0.156 | <.001 | -0.171 | <.001 |
| cv of DBP |  |  |  |  |  |  |
|  | mean of DBP | 1506 | 0.038 | .999 | -0.028 | .999 |
|  | max of DBP | 1506 | 0.348 | <.001 | 0.242 | <.001 |
|  | min of DBP | 1506 | -0.301 | <.001 | -0.339 | <.001 |
| sd of PP |  |  |  |  |  |  |
|  | cv of PP | 1506 | 0.966 | <.001 | 0.951 | <.001 |
|  | mean of PP | 1506 | 0.133 | <.001 | 0.159 | <.001 |
|  | max of PP | 1506 | 0.446 | <.001 | 0.438 | <.001 |
|  | min of PP | 1506 | -0.224 | <.001 | -0.144 | <.001 |
| cv of PP |  |  |  |  |  |  |
|  | mean of PP | 1506 | -0.093 | .01 | -0.116 | <.001 |
|  | max of PP | 1506 | 0.236 | <.001 | 0.178 | <.001 |
|  | min of PP | 1506 | -0.440 | <.001 | -0.410 | <.001 |

Abbreviations: SBP, systolic blood pressure; DBP, diastolic blood pressure; PP, pulse pressure; baPWV, brachial-ankle pulse wave velocity; sd, standard deviation; cv, coefficient of variation.

^a^ Spearman’s coefficients were given.

**Figure S1:** Study period and definition of two phases.


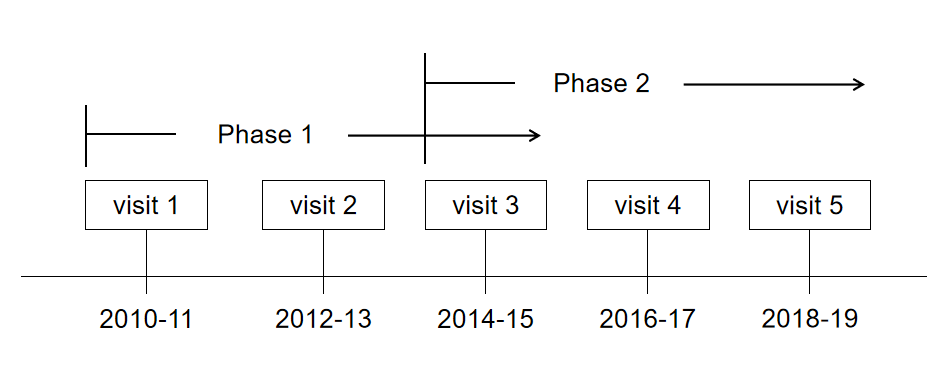


**Figure S2: The regression lines of BPV indexes at phase 1 and the subsequent artery stiffness level.**


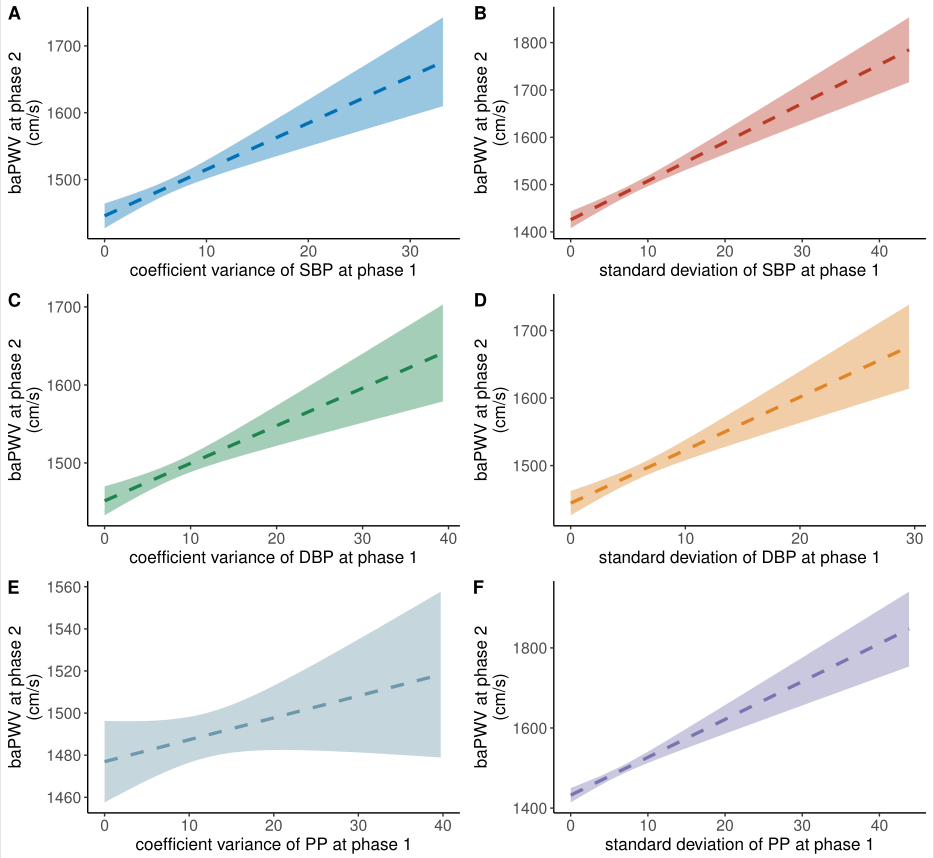


A: CV of SBP and baPWV; B: SD of SBP and baPWV; C: CV of DBP and baPWV;

D: SD of DBP and baPWV; E: CV of PP and baPWV; F: SD of PP and baPWV.

Abbreviations: SBP, systolic blood pressure; DBP, diastolic blood pressure; PP, pulse pressure; baPWV, brachial-ankle pulse wave velocity; SD, standard deviation; CV, coefficient of variation.
